# Clinical Approach to Manage Gastrointestinal Bleeding with a Left Ventricular Assist Device (LVAD)

**DOI:** 10.7759/cureus.6341

**Published:** 2019-12-10

**Authors:** Irfan Ahsan, Aniqa Faraz, Asif Mehmood, Waqas Ullah, Ali R Ghani

**Affiliations:** 1 Internal Medicine, Geisinger Health System, Danville, USA; 2 Internal Medicine, University of Buffalo, Buffalo, USA; 3 Internal Medicine, Geisinger Medical Center, Danville, USA; 4 Internal Medicine, Abington Hospital - Jefferson Health, Abington, USA; 5 Cardiovascular Medicine, Saint Louis University, Saint Louis, USA

**Keywords:** gastrointestinal bleeding, end-stage heart failure, left ventricular assist device (lvad)

## Abstract

Left ventricular assist devices (LVADs) are an exceedingly important form of mechanical support for patients with end-stage heart failure. LVADs can be utilized both as a bridge to cardiac transplant and also as a definitive treatment. However, a few complications are associated with LVAD placement, the most common and cumbersome of which is gastrointestinal (GI) bleeding with an incidence of about 30%. These bleeding events often require transfusion therapy, but they are rarely fatal.

The etiologies of GI bleeding following LVAD are multifactorial and include unstable hemodynamics, an acquired von Willebrand factor (vWf) deficiency, impaired platelet aggregation, and activation of fibrinolytic systems. The treatment of choice in LVAD implantation-associated GI bleeding is endoscopy, which plays a vital role in both its diagnosis and management. Even so, its effectiveness in controlling post-LVAD implantation GI bleeding is still poorly ascertained. In this article, we will review the use of medication and alterations in the LVAD setting to prevent the occurrence of GI bleeding, as well as the findings of previously reported literature on LVAD implantation-associated GI bleeding.

## Introduction and background

Heart failure is a progressively debilitating and chronic disease afflicting approximately 5.8 million adults in the United States (US) [1]. It has been singled out as an epidemic and is a progressive, debilitating health care problem with significant mortality and morbidity, especially in patients above 65 years [2]. It carries a mortality of around 50% in the next five years, thereby surpassing many malignancies [3]. The continuous rise in the number of patients with progressive heart failure has been due to prolonged survival from improved cardiovascular interventions and advancements in medical therapy. The mainstay of management has mostly been medical over the past years, but patients who are considered refractory to medical therapy are put on waiting lists for heart transplantation. A heart transplant is the gold standard treatment for end-stage heart failure; however, due to the limited availability of donor hearts, it is difficult to perform a transplant on every patient with end-stage heart failure. According to statistics, only 2,000 transplants are performed every year as a consequence of the donor shortage throughout the United States [4]. Once readmissions for heart failure exacerbations seem to recur, the prognosis tends to get worse [5-6]. The first-ever mechanical circulatory device was implanted in 1963 by DeBakey in a patient who had cardiac arrest after aortic valve replacement [7]. In the 1980s, ventricular assist devices (VADs) were first approved by the Food and Drug Administration (FDA) to be used as a bridge to transplant [8]. Since the Randomized Evaluation of Mechanical Assistance for the Treatment of Congestive Heart Failure (REMATCH) trial in 2001, left ventricular assist devices (LVADs) are not only used as a bridge to transplant but also as a destination therapy for myocardial recovery among those who are not suitable candidates or for whom a timely transplant is not available [9-10]. LVAD-related complications occur in 60% of patients within six months, and by two years, the majority of patients have experienced a major adverse event [11]. Gastrointestinal (GI) bleeding is a major cause of morbidity after LVAD implantation and its incidence has increased from 5% in 2005 to around 10% in 2010 [12]. Traditionally started as a pulsatile flow (PF) LVADs (which had a larger size and were more prone to thrombosis), continuous flow (CF) LVADs have become the standard of care with over three times the increased risk of GI bleeding. This ultimately leads to an increase in the length of hospital stay, the cost burden on health care resources, and hospital readmission rates [10, 12]. The purpose of this article is to review the literature focusing on the diagnosis and management of gastrointestinal bleeding in patients with LVAD from an internist's point of view.

Clinical presentation and risk factors

Patients with LVAD-associated GI hemorrhage most commonly present with hematemesis, melena, and rectal bleeding. Some patients may be otherwise asymptomatic, while others may complain of fatigue, weakness, or dizziness. Hemodynamic instability may occur in a few cases. These symptoms often develop five months subsequent to LVAD placement but may also occur immediately or long after LVAD placement. Laboratory analysis may frequently show anemia, a raised international normalized ratio (INR), and a decrease in the platelet count.

GI bleeding following LVAD placement has various risk factors and is most commonly seen in elderly males. Other risk factors include a previous history of pre-LVAD GI bleed, antiplatelet and vitamin K antagonist use, right ventricular dysfunction, and post-LVAD ejection fraction > 30% [13].

Pathophysiology and etiology

The pathophysiology of GI bleeding secondary to LVAD implantation is still controversial. One of the mechanisms to explain the increased risk of bleeding is endothelial dysfunction from non-pulsatile blood flow, which was more commonly observed in patients supported by CF-LVADs than PF-LVADs. It was discovered that there was a loss of physiologic pulsatility which is associated with the normal cardiac contraction cycle in patients treated with CF-LVADs, consequently causing a narrowed arterial pulse pressure and leading to a decrease in the aortic valve opening [14]. 

Another mechanism proposed is acquired von Willebrand disease from increased degradation of the von Willebrand factor (vWF) impaired platelet aggregation, leading to increased bleeding [15-16]. Acquired vWD was first described in 2003 to explain arteriovenous malformations (AVMs) in severe aortic stenosis (Heyde’s syndrome). This pathophysiology is believed to be somewhat similar to AVM bleeds in CF-LVADs [17]. It is hypothesized that vWF has an important role in the regulation of angiogenesis [18]. In 2011, a study published by Starke et al. recruited mice as subjects and demonstrated that a lack of vWF in the Weibel-Palade bodies of endothelial cells led to increased angiogenesis and neovascularization in those mice subjects [19]. Therefore, it can be concluded that a deficiency of vWF leads to an increased number of endothelial cells and cytokines which leads to an increased rate of angiogenesis and subsequently to the development of arteriovenous malformations. 

Some authors also attribute GI bleeding to the decreased amount of high-molecular-weight multimers due to the mechanical forces of the LVAD device, leading to ineffective thrombosis and platelet aggregation [20]. This was shown in a study performed by Hayes et al. in which 37 patients had undergone CF-LVAD implantation [21].

The use of both warfarin and aspirin has also been implicated as the cause of increased bleeding in patients with CF-LVAD as compared to aspirin only in PF-LVAD patients. These patients required anticoagulation because of the increased risk of pump thrombosis. However, some studies have shown that a decrease in INR (1.5 - 2.5 range) could be achieved without posing a risk for thromboembolism [22]. Furthermore, a few studies have suggested that there might be a genetic predisposition to the development of bleeding tendencies and thromboembolism in LVAD patients. It is suggested that, in patients who are sensitive to warfarin, there may be rare polymorphisms in CYP2C9 and vitamin K epoxide reductase complexes, leading to bleeding and thromboembolism [23]. However, this hypothesis does necessitate further studies for its justification.

In observational studies, CF-LVAD was associated with a 10-fold increased risk of Gl bleeding as compared to PF-LVAD [15]. Similarly, in a cohort of 112 patients, 39% of the patients had GI bleeding, and endoscopy was able to identify the bleeding source in 57% of the patients [16].

Other mechanisms that could lead to GI bleeding in patients of LVAD include impaired platelet aggregation, activation of fibrinolytic systems, and nonsteroidal anti-inflammatory drug (NSAID) use. It was observed that the majority of LVAD recipients had impaired platelet aggregation which normalized after a heart transplant [24]. This impairment in platelet function occurs due to an increased sensitivity of the platelets to shear stress [25].

**Figure 1:**
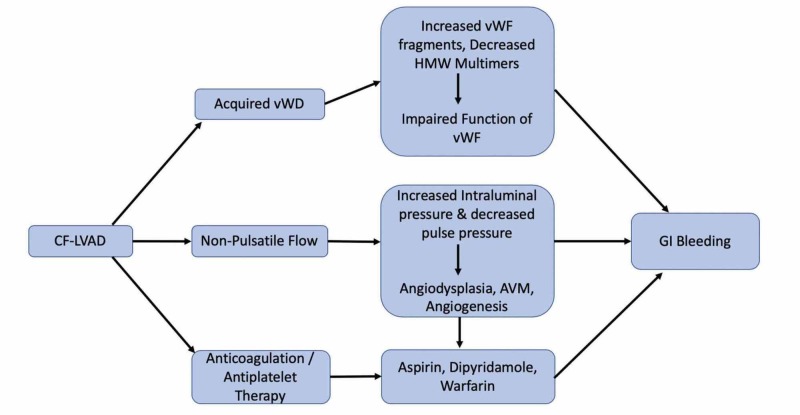
Algorithm on the pathophysiology of gastrointestinal bleeding AVM: arteriovenous malformation; CF-LVAD: continuous-flow left ventricular assist device; GI: gastrointestinal; HMW: high-molecular-weight; vWD: von Willebrand disease; vWF: Von Willebrand factor

Patients undergoing LVAD implantation have activation of fibrinolytic systems, especially elderly patients with end-stage cardiac failure. The cause of this increased fibrinolysis is most likely a result of a preexisting state of inflammation [26]. It has been observed that the majority of post-LVAD placement bleeding episodes occur during the first year [27] and that d-dimer and fibrinogen levels peak at one month and return to normal a year after of LVAD implantation [28]. The pathophysiology of GI bleeding has been discussed in Figure 1.

The upper GI tract, lower GI tract, and small bowel account for 48%, 22%, and 15% of GI bleeding episodes, respectively [29]. The majority of bleeding episodes in LVAD patients are secondary to underlying AVMs and they are usually not life-threatening. If the bleeding is brisk, it is less likely related to an AVM and the patient needs an emergent scope to rule out other etiologies of gastrointestinal bleeding. The majority of bleeding episodes do get resolved in an average of four days and usually require an average of four packed red blood cell units [30-32]. Table 1 discusses the various studies which highlighted the cause of GI bleeding.

**Table 1:** Summary of All Studies Identifying the Source of GI Bleeding ^a^VentrAssist^™^ (Ventracor Ltd., Sydney, Australia) ^b^Jarvik 2000® (Jarvik Heart, Inc., New York, NY) ^c^HeartWare HVAD® (HeartWare, Framingham, MA) ^d^HeartMate II^™ ^LAVD (Thoratec Corp., Pleasanton, CA) ^e^HeartMate® XVE (Thoratec Corp., Pleasanton, CA) AVM: arteriovenous malformation; HM-XVE: HeartMate™ XVE; LVAD: left ventricular assist device

S. No	Author	Year of Study	Details of LVAD placement	Total Incidence	Gastritis	Gastric Ulcer	AVM	Diverticulitis	Colitis	Colonic Polyp	Colonic Ulcer	Other	Unknown
1	Hayes et al. [21]	2010	^a^VentrAssist ™ : 20%, ^b^Jarvik 2000^®^: 40%, ^c^HeartWare^®^: 40%	13.9%			60.0%				20.0%		20.0%
2	Demirozu et al. [13]	2011	Continuous-flow ^d^HeartMate II ™ LVAD	19.0%	31.3%		31.3%	18.8%	3.2%	3.2%		12.5%	
3	Aggarwal et al. [33]	2012	Continuous-flow ^d^HeartMate II ™ LVAD	22.8%	30.4%	8.7%	21.7%			4.3%	13.0%	13.0%	8.7%
4	Kushnir et al. [34]	2012	Pulsatile ^e^HM-XVE: 27.3%; continuous-flow ^d^HeartMate II ™ : 72.7%	34.8%		28.2%	30.8%		5.1%	5.1%			30.8%
5	Wever-Pinzon et al. [11]	2013	Continuous-flow ^d^HeartMate II ™ LVAD	17.2%	8.7%		61.0%	8.7%	4.3%	8.7%		8.7%	

## Review

GI bleeding is a dreaded complication of LVAD implantation which can potentially lead to hemodynamic instability and eventually life-threatening hemorrhagic shock. The management of post-LVAD GI bleeding requires urgent evaluation with a multidisciplinary approach. Managing the anticoagulation in these patients can be challenging and comes at the risk of pump thrombosis versus life-threatening hemorrhage. The role of a clinician is important in evaluating such patients who are at high risk of life-threatening bleeding. Furthermore, the risk stratification of these patients who are at risk of recurrence and readmissions is required. In patients with no obvious bleeding but a significant drop in hemoglobin, LVAD thrombosis or hemolysis is a crucial differential to be considered and requires urgent intervention. High serum lactate dehydrogenase (LDH) can play a vital role in differentiating hemolysis from bleeding [35]. The management strategy relies on the patient hemodynamics, severity of bleeding, coexisting coronary artery disease, the availability of state-of-the-art therapeutic options, and other indications of anticoagulation (e.g., the history of the thromboembolic phenomenon). Initial resuscitation by intravenous crystalloids and blood products, followed by noninvasive diagnostic modalities (e.g., computed tomography angiography) and invasive techniques (e.g., esophagogastroduodenoscopy, video capsule endoscopy, tagged red blood cell (RBC) scan, mesenteric angiogram, single balloon enteroscopy, deep balloon-assisted enteroscopy, and colonoscopy) are the major tools in the clinical approach.

Management

Antithrombotics are the cornerstone therapy in the outpatient management of LVAD. In addition to aspirin, warfarin is the anticoagulant of choice, while direct thrombin inhibitors (e.g., dabigatran) have failed to ascertain their usefulness in these patients [36]. Dabigatran was studied in a prospective randomized study in which 30 patients were randomized to either dabigatran or phenprocoumon (vitamin K antagonist). This trial was prematurely discontinued due to the increased thromboembolic phenomenon observed in half of the patients (four out of eight) receiving dabigatran [36]. On warfarin therapy, the target INR is 2.0 - 3.0 in LVAD patients [30]. LVAD patients with previous deep venous thrombosis, pulmonary embolism, and inherited thrombophilia have a higher INR goal (2.5 - 3.5) [31]. Similarly, patients with atrial fibrillation and/or history of stroke have an INR goal of 2.5 - 3.0. Higher INR goals require repetitive testing and are associated with bleeding and thromboembolic complications with an event rate of 0.06 per patient-year for GI bleeding [31]. At a follow-up of one year, only one-third of patients are without bleeding or thromboembolic complications. At the time of bleeding, the average INR was found to be approximately 3.3 [32]. Ultimately, the management relies upon the individual clinician and the patient [37].

**Figure 2:**
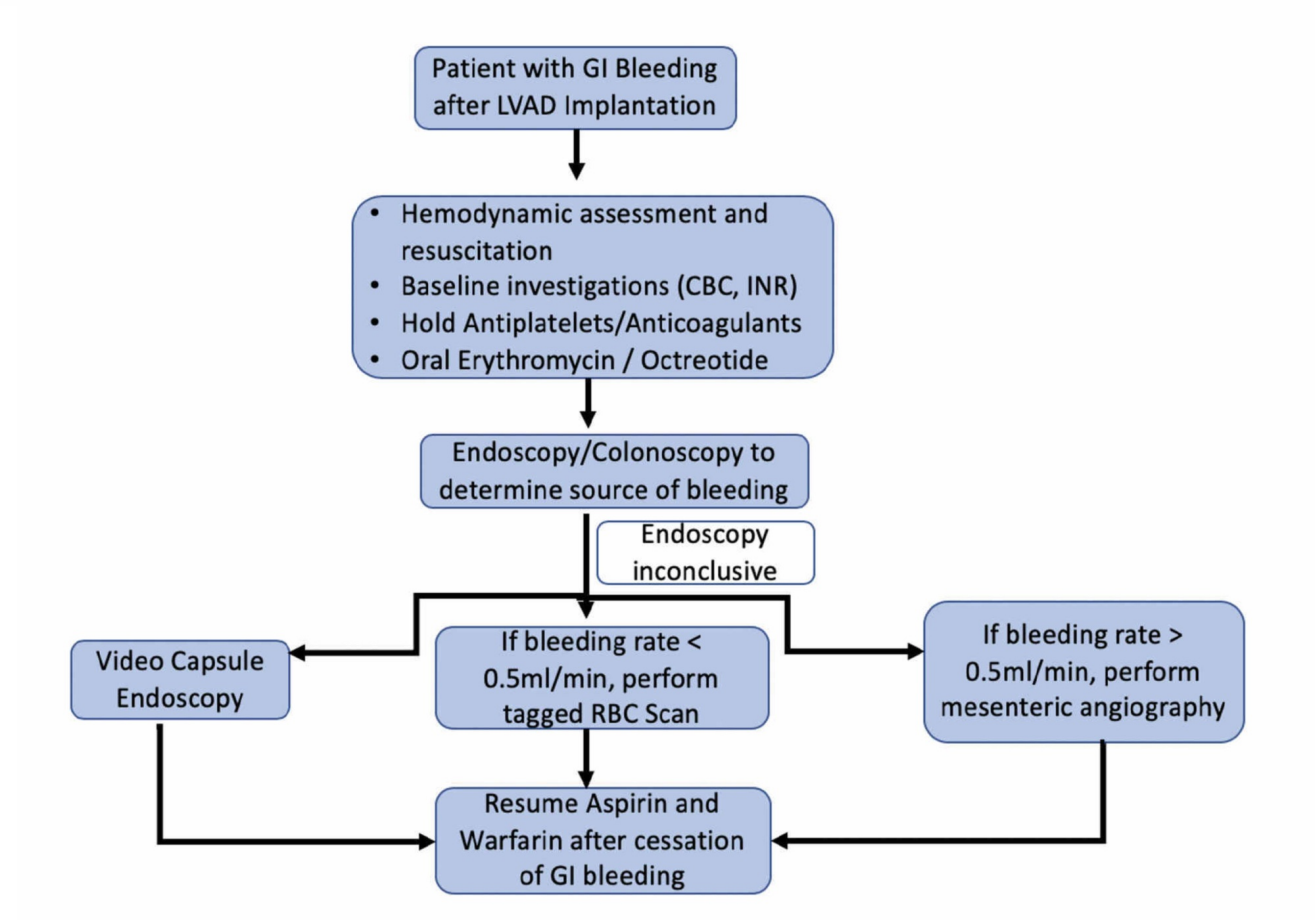
Flowsheet outlining the management approach to GI bleeding in patients with LVAD placements CBC: complete blood count; GI: gastrointestinal; INR: international normalized ratio; LVAD: left ventricular assist devices; RBC: red blood cells

LVAD patients with gastrointestinal hemorrhage require a multidisciplinary and comprehensive approach. A detailed history and examination should be performed, along with initial assessment and hemodynamic resuscitation. As mentioned earlier, NSAID use is a predisposing factor to the development of GI bleeding; hence, their use should be enquired in the patient history. Physical examination should include the assessment of LVAD function by the cardiology team and a gastroenterology consult should be taken in the Emergency Department, after which intensive care admission should be assessed. Investigations should include a complete blood count, metabolic and hepatic panels, and should also focus on INR, fibrinogen, and d-dimer levels. However, the decision of withholding antiplatelet and anticoagulation therapy needs to be discussed with the Cardiology Department, keeping in mind the risk of thrombosis in the immediate future. A schematic outline of the management approach has been summarized in Figure 2.

Mild to Moderate Bleeding

The majority of gastrointestinal bleeds in LVAD patients are not life-threatening but are a cause of increased morbidity and recurrent hospitalizations. For patients with mild to moderate bleeding, the approach should be to stop the anticoagulation and perform an endoscopy. Endoscopy is an extremely useful modality for the diagnosis and treatment of GI bleeding, but it can yield false-negative results by stopping the occult bleed. Moreover, endoscopy is non-diagnostic in over two-thirds of the patients [16]. After the source of bleeding is found and treated accordingly, aspirin should be withheld and warfarin should be restarted with a lower INR goal. Maintaining a balance between bleeding and thromboembolism with close outpatient follow-up is a decision that rests with the physician and patients, based on individualized risk factors [37]. Holding anticoagulation for two to four weeks after GI bleeding may lead to an increased risk of thromboembolic events. In these patients who present with hematochezia or an occult bleed, colonoscopy after bowel preparation is used to diagnose the cause of GI bleeding. In the case of a suspected small intestinal angiodysplastic lesion, a push enteroscopy is required to evaluate the proximal jejunum. About 29% - 90% of LVAD patients who had GI hemorrhage benefited from push enteroscopy, thereby decreasing their risk of future rebleeding and readmission [16]. If colonoscopy fails to disclose the etiology, then performing an esophagogastroduodenoscopy (EGD) is the next best step. According to the American College of Gastroenterology guidelines, the patients with an upper GI bleed should undergo endoscopy within 24 hours, following resuscitative efforts to optimize hemodynamic parameters [30]. When both these measures fail, video capsule endoscopy should be performed as it has been shown to have a high bleeding detection rate (about 40% of cases, mostly confined to the proximal small intestine) [38]. However, video capsule endoscopy is not therapeutic and may result in an increased hospital stay of the patient. In an active overt GI bleed, a tagged RBC scan and angiography are the other modalities to consider. The bleeding rate needs to be determined, i.e, if the bleeding rate is 0.1 - 0.5 ml/min, then a tagged RBC scan can be appropriate; however, for > 0.5 ml/min bleeding, angiography with suspected possible angiographic intervention is done [39].

Severe Bleeding

In patients who are hemodynamically unstable or have profuse bleeding, immediate resuscitative efforts to maintain hemodynamic stability and prophylactic intubation should be performed. All anticoagulant and antiplatelet drugs should be stopped and fluid resuscitation should be initiated. Immediate consults from gastroenterologists or concerned specialists should be obtained. Vitamin K is given in severe cases of bleeding when extended time is expected to be off anticoagulation. However, once the patient is stabilized, warfarin should be restarted immediately. Restarting warfarin anticoagulation can prove to be difficult due to excess vitamin K levels in the blood. The decision to reverse anticoagulation should be made after involving the LVAD team (heart-failure specialist and cardiothoracic surgeon) and reviewing patient parameters like PT/INR (prothrombin time/international normalized ratio) and hemodynamic status as to give vitamin K or fresh frozen plasma. 

Controversial Medical Therapies

Several medical therapies have been studied which have not yet been inculcated into clinical practice. Oral erythromycin (250 mg) 30 minutes before endoscopy may improve diagnostic yield by removing clots [40]. The role of nasogastric lavage has been controversial. In previous studies, a positive lavage was useful in finding the high-risk lesion and it has been associated with the earlier endoscopy, but it was not associated with a change in clinical outcomes [41]. The somatostatin analog, octreotide, is an effective therapy to control GI bleeding, especially when due to angiodysplasia [42]. By splanchnic vasoconstriction, platelet aggregation, and angiogenesis inhibition, it leads to the reduction in the requirement of blood units and hospital admissions after LVAD implantation-associated GI bleeding. A retrospective review performed by Hayes et al. successfully treated five patients with GI bleeding using octreotide, adrenaline infusion, and a reduction in pump flow [21]. However, a larger case series showed that octreotide did not have a noteworthy effect on the rate of recurrent GI bleeding. Thalidomide has also been used in various small studies because of its angiogenic properties [43]. Patients with LVAD have a high factor VIII and ristocetin cofactor activity due to inflammation; however, in patients with cardiac etiology, only 10% have responded to desmopressin [16]. A recent study by Fischer et al. demonstrated the use of vWF concentrate, devoid of factor VIII, in a CF-LVAD patient with severe GI bleeding. The use of vWF concentrate helped stabilize hemoglobin and effectively stopped bleeding without increasing the risk of thromboembolism [44]. The management of GI bleeding has been summarized in Figure 2. These studies warrant a need for further research in the management of GI bleeding secondary to LVAD placement, with emphasis on individual anticoagulant therapy. Table 2 discusses the incidence of GI bleeding after LVAD implantation in recent observational studies.

**Table 2:** Incidence of GI Bleeding After LVAD Implantation in Recent Observational Studies CF-LVAD: continuous-flow left ventricular assist device; GI: gastrointestinal; LVAD: left ventricular assist device; N/A: not available

Study Author	Year of Publication	Major Question	Major Outcome of The Study	Treatment
Aggarwal et al. [33]	2012	To identify incidence, etiology, and management of GI bleed in LVAD patients	The incidence was found to be 22.8% in the destination therapy population. Multiple factors are responsible for GI bleeding in LVAD patients	Stopping anticoagulation therapy, reduced the speed of LVAD and octreotide treatment
Akhter et al. [45]	2015	To find incidence and causes of hospital readmissions after LVAD implantation	GI bleeding and LVAD-related infections were the major causes of readmissions. These readmissions were found to have no impact on long-term survival.	N/A
French et al. [46]	2013	When is the highest rate of GI bleeding following LVAD implantation?	The highest hazard by GI bleeding was found to be early post-implantation, stressing the importance of follow-up immediately after implantation.	N/A
Jabbar et al. [47]	2015	To identify the incidence, recurrence, and predictors of GI bleeding and impact of endoscopy in LVAD patients.	GI bleeding is common, especially from the upper GI tract. An upper endoscopy can identify lesions in 50% of patients. For management, a reduction in pump speed is an effective strategy.	Stopping anticoagulation/antiplatelet therapy; endoscopy
Yavar et al. [48]	2017	Sex-related bleeding complications after CF-LVAD	Females had a 60% higher hazard of bleeding than males with significant morbidity encountered from mucosa (including vaginal) bleeding. Future large device studies should be inclusive of sex-specific outcomes.	Admission and blood transfusion

## Conclusions

With the increased longevity of heart failure patients, LVAD has become the standard of care in contemporary practice as a bridge to transplant or destination therapy. Due to the paucity of data, post-LVAD bleeding poses a challenge to the anticoagulation of such patients who are at high risk of pump thrombosis. A multidisciplinary team-based approach is vital in the prompt diagnosis and treatment of GI bleeding in LVAD patients. Further studies are required to define the predictors of GI bleeding so that high-risk patients can be risk-stratified to decrease morbidity and mortality.
